# Development and quality evaluation of high-protein gluten-free pasta formulations

**DOI:** 10.1038/s41598-025-12336-5

**Published:** 2025-07-26

**Authors:** Sayed Mostafa, Shymaa M. Ata, Ahmed M. S. Hussein, Ahmed A. Zaky

**Affiliations:** 1 Department of Food Technology, Food Industries and Nutrition Research Institute, National Research Centre, Dokki, Cairo, 12622 Egypt; 2 Home Economics Department, Faculty of Specific Education, Menoufia University, Menoufia, 32951 Egypt

**Keywords:** Gluten-free diet, Oat flour, Xanthan gum, Guar gum, Physicochemical, Sensory attributes, Biophysics, Proteins

## Abstract

The production of high-quality gluten-free pasta remains a persistent challenge, as it is the only effective dietary treatment for individuals with celiac disease. This study aimed to develop and evaluate high-protein gluten-free pasta (GFP) with acceptable quality. To increase GFP quality, response surface methodology (RSM) was employed to identify the optimal ratios of xanthan gum (XG) and guar gum (GG) as binding agents. The optimal concentrations of these hydrocolloids, determined as 2.00% XG and 0.75% GG, were used to formulate a GFP based on rice flour (RF), oat flour (OAF), and lentil flour (LF) at varying concentrations. The impacts of different formulations on pasting behavior and functional properties were assessed. The final products were evaluated for their physicochemical properties, hardness, phenolic content, protein digestibility, cooking quality, and sensory attributes. Notably, compared with RF, OAF resulted in a decreased peak viscosity (684.10 to 488.00 cP) and holding strength (548.90 to 357.60 cP), with a higher pasting temperature (59.10 to 63.40 °C) and breakdown (99.18 to 130 cP). LF negatively affected these parameters, which decreased proportionally with increasing LF replacement levels. Compared with RF, OAF resulted in significantly lower swelling power (6.88 to 4.96 g/g) and higher oil holding capacity (157 to 1.80 g/g). Higher LF levels reduce raw pasta hardness but positively impact the phenolic content and protein digestibility of cooked pasta. Sensory evaluations revealed that LF improved color, taste, and overall acceptability. This study contributes to the development of high-quality GFP, addressing both technological and nutritional challenges in gluten-free pasta production.

## Introduction

Celiac disease is a chronic autoimmune disorder that primarily affects the small intestine and is triggered by an adverse reaction to gluten, a protein found in wheat, barley, and rye^1,2^. This condition results in inflammation and damage to the intestinal villi, leading to malabsorption of nutrients, which can cause symptoms such as diarrhea, weight loss, and fatigue^3^. The disease is hereditary and affects approximately 1 in 100 people worldwide, although only approximately 30% are properly diagnosed^4^.

In addition to its gastrointestinal impacts, celiac disease can have broader health implications, including effects on the skeletal system, such as osteoporosis, and an increased risk of other autoimmune disorders^4^. The only effective treatment for celiac disease is a strict, lifelong gluten-free diet, which also benefits individuals with nonceliac gluten sensitivity^5^.

Pasta, a staple food worldwide, poses a challenge for those with gluten sensitivity because of its high gluten content. Gluten-free alternatives often lack sensory appeal and nutritional value, highlighting the need for innovative formulations that meet both dietary needs and health standards^6,7^. Recent research has focused on enhancing the nutritional profile of pasta by incorporating unconventional ingredients, driven by consumer demand for functional foods^2,8^.

Legume flours, such as lentil flour (LF), are promising solutions for improving gluten-free products. Lentils are rich in protein, dietary fiber, and essential minerals, and they provide beneficial bioactive compounds^9,10^. The incorporation of LF into gluten-free pasta formulations, particularly when combined with rice and oat flours, can enhance nutritional value and provide a more sustainable alternative to traditional pasta^11,12^. This approach not only addresses the dietary needs of individuals with gluten sensitivities but also aligns with the growing preference for plant-based protein sources and environmentally sustainable food options^13,14^.

The incorporation of composite flours in gluten-free product development has gained attention, particularly in developing regions such as North Africa, due to both economic and nutritional motivations^15^. In this context, hydrocolloids play a crucial role in compensating for the absence of gluten, offering structural and rheological functions essential for acceptable dough and final product quality^16^. Among these, xanthan gum and guar gum are widely utilized for their complementary properties; xanthan provides thermal and pH stability with excellent thickening and gas retention capacities, while guar enhances viscosity and hydration^17^. However, individual hydrocolloids often fall short in fully replicating gluten’s viscoelasticity, making binary combinations more effective^18^. Studies have shown that xanthan–guar blends produce synergistic effects, such as increased volume, reduced staling, and improved crumb structure in gluten-free products^19^. These interactions result in bi-continuous matrices and thermo-reversible gels, crucial for texture improvement^20^. Therefore, the strategic use of gum combinations, especially xanthan and guar, is fundamental in advancing the technological quality of gluten-free products^18^.

Although numerous studies have aimed to improve the quality of gluten-free pasta, most have investigated hydrocolloids either individually or at fixed concentrations^21–24^. In contrast, the novelty of the current study lies in its use of a full factorial design (5 × 5) to systematically evaluate the interactive effects of xanthan gum (XG) and guar gum (GG) at varying concentration levels (0.0–2.0 g/100 g RF) on the texture properties, particularly the hardness, of gluten-free pasta. This comprehensive approach enables a deeper understanding of the synergistic or antagonistic effects between these two hydrocolloids, which has not been fully explored in previous literature. Despite growing demand, gluten-free pasta often falls short in nutritional and sensory quality. A comparative study of 235 gluten-free and 349 conventional pasta products on the Italian market revealed that gluten-free varieties had significantly lower protein and fiber content^25^, as well as inferior texture and physical properties^26^ and reduced sensory appeal^27^. These limitations highlight the need for formulation strategies that enhance the nutritional profile and quality characteristics of gluten-free pasta. Therefore, the current study aims to identify the ideal concentration of hydrocolloids (xanthan gum and guar gum) using RSM to achieve the maximum hardness in dry gluten-free pasta (GFP). This study also explored the production of GFP when rice-oat flour was combined with various proportions of lentil flour, examining how these different mixtures influence the physicochemical and nutritional characteristics of the final product.

## Materials and methods

### Materials

Three raw gluten-free raw materials were selected: rice (*Oryza sativa*), oat (*Avena sativa*), and lentil (*Lens culinaris* L.). These materials were purchased from a local market in Cairo, Egypt. Xanthan gum (XG) powder, which is food grade, was obtained from LANDOR Trading Co., whereas guar gum (GG) pure powder was obtained from NOW FOODS, both of which are from the local market in Egypt. Other chemicals and reagents were obtained from Sigma‒Aldrich, Saint Louis, USA.

### Methods

#### Preparation of rice and oat flour

The grains were cleaned to remove foreign matter, and each type was ground separately using an electric grinder (MIENTA Super Blender, Model BL-721) to pass through a 300-mesh sieve. The flours were then sealed in polyethylene bags and stored at 4 °C until further^28^.

#### Preparation of lentil flour

One kilogram of legume seeds (lentils) was cleaned of dirt and other foreign materials, such as stones and sticks. The seeds were then soaked in 2 L of tap water for 12 h. After soaking, the seeds were ground using a mixer (MIENTA super blender, Model BL-721) and dried in a cabinet dryer at 120 °C for 90 min. During the drying process, the ground seeds were stirred at 30-minute intervals to ensure uniform drying. The dried lentils were ground and sieved to pass through a 300-mesh sieve. The resulting flour was packaged in sealed polyethylene bags until use.

#### Experimental design for the optimization of binding agents

The response surface approach was used to determine the optimum concentration of hydrocolloids that achieves the highest dry pasta hardness. The experiment was conducted on a control sample (100% rice flour), and both xanthan and guar gums from 0 to 2% (Table 1) were used as full factorial experimental design of the aforementioned hydrocolloids. These variations were assessed to identify the ideal mixture for improving dry pasta hardness.

**Table 1:** Central composite design for Xanthan gum and Guar gum concentrations.

Samples	Binding agents (g/100 g rice flour)		Hardness values (*N*)
	Xanthan gum	Guar gum	
1	0.0	0.0	9.00
2	0.0	0.5	11.54
3	0.0	1.0	17.27
4	0.0	1.5	27.17
5	0.0	2.0	35.8
6	0.5	0.0	12.75
7	0.5	0.5	18.32
8	0.5	1.0	26.59
9	0.5	1.5	35.87
10	0.5	2.0	42.43
11	1.0	0.0	13.27
12	1.0	0.5	22.81
13	1.0	1.0	36.77
14	1.0	1.5	39.28
15	1.0	2.0	41.28
16	1.5	0.0	19.27
17	1.5	0.5	26.8
18	1.5	1.0	33.12
19	1.5	1.5	40.67
20	1.5	2.0	42.18
21	2.0	0.0	31.26
22	2.0	0.5	34.81
23	2.0	1.0	35.87
24	2.0	1.5	41.72
25	2.0	2.0	43.38

#### Pasta mixture blends

In the preliminary phase of the study, pasta samples were formulated using different concentrations of XG and GG to evaluate their impact on the hardness of dry pasta that was prepared from RF. Response surface methodology (RSM) was employed to identify the optimal level of these hydrocolloids that maximizes pasta hardness. Based on the RSM data, the combination of 2.00% XG + 0.75% GG was found to yield the highest hardness value. This optimal gums ratio was subsequently applied in all the composite formulations involving rice, oat, and lentil flours, as presented in Table 2.

**Table 2:** Gluten free pasta formulas.

Formulas	Ingredients (g)				
	Rice flour	Oat flour	Lentil flour	Xanthan gum	Guar gum
C1	100	-	-	2.00	0.75
C2	-	100	-	2.00	0.75
M1	50	50	-	2.00	0.75
M2	45	45	10	2.00	0.75
M3	40	40	20	2.00	0.75
M4	35	35	30	2.00	0.75

C1 = 100% rice flour, C2 = 100% oat flour, M1 = 50% rice flour + 50% oat flour, M2 = 45% rice flour + 45% oat flour + 10% lentil flour, M3 = 40% rice flour + 40% oat flour + 20% lentil flour, M4 = 35% rice flour + 35% oat flour + 30% lentil flour.

#### Production and drying procedures

The gluten-free pasta dough was prepared according to the method described by^29^. The dry ingredients were thoroughly mixed to ensure uniformity. The mixture was then transferred to a mixing bowl and blended until a cohesive dough formed. The dough was shaped into a ball, covered with plastic wrap, and allowed to rest for 30 min. After resting, it was hand-kneaded for one minute, divided into portions of approximately 100 g, and processed via a pasta machine (Philips Pastamaker HR2357/05 Machine Corporation, Italy). The pasta was air-dried for 4 h. The mixture was subsequently transferred to a dehydrator in a cabinet and dried to a moisture content of 12% ± 1 at 70 °C. Once dried, it was cooled to room temperature (25 °C ± 2) and stored in plastic bags until testing.

### Analytical methods

#### Approximate analysis and mineral content

The samples were subjected to chemical analysis for moisture and ash contents, following the AACC^30^ International methods 44 − 15 (Moisture‒Air Oven Method) and 08 − 01 (Ash‒Basic Method), respectively. The analysis of lipids and crude protein (Nx5.7) was conducted in accordance with the procedures outlined in^31^. Lipids were extracted via a Soxhlet apparatus with N-hexane as the solvent. The nitrogen-free extract (NFE) was calculated by difference. The mineral contents (Fe, Zn, K and Mg) were determined as described by^2^.

#### Cooking quality

Pasta cooking quality was assessed following the method established by the^30^. The optimum cooking time (OCT) refers to the duration needed for the opaque center of the pasta to vanish when it is gently pressed between two glass plates post cooking. The cooking yield (CY) was calculated as the percentage weight increase. The solids content in the cooking water was measured by drying at 105 °C overnight. Cooking loss (CL) was expressed as a percentage derived from the solid weight relative to the initial dry matter. To determine the swelling index (SI), the water displacement of the cooked pasta is divided by that of an equivalent amount of uncooked pasta. Nitrogen loss (NL) was measured via the Kjeldahl method as approved by the AACC^30^.

#### Hardness

The hardness (N) of dry gluten free pasta (Length: 40 mm and thickness: 12 mm) was determined in triplicate via CT3™ texture analyzer **(**Brookfield) according to^15^. The computer was set for TestWorks software, and an appropriate test was selected (Compression): a test speed of 2.50 mm/s and a load cell of 10,000 g in one cycle using TA-7 Knife edge probe for dry pasta with a depth of 10 mm.

#### Functional properties

The water-holding capacity (WHC) of prepared pasta formulas was determined according to the AACC method 56–20^30^. The oil-binding capacity (OBC) of pasta formulas was measured following the method of^32^. The WHC and OBC of the flours were calculated on a dry basis (db). The swelling power was adapted from^33^. The sample (0.5 g) was mixed with 20 ml of water, and the mixture was heated from 30 °C to 90 °C°C for 30 min. The sample–water mixture was then weighed, and more water was added until the weight of the mixture reached 25 g. Centrifugation at 11,000 × g for 15 min was performed to separate the solid residue and the supernatant. The swelling power was determined via the following formula:

$$\text{Swelling power (g/g)}=\frac{\text{Wet residue weight }(\text{g}) -\text{ dry defatted }(\text{g})}{\text{dry defatted }(\text{g})}$$

#### Pasting properties

The pasting properties of the prepared formulas were measured via a rheometer (RheoLab QC, Anton Paar, GmbH, Graz, Austria) in accordance with^34^. The samples were evaluated based on 3.5 ± 0.01 g of sample adjusted to 14% moisture content. The quantity of water added was 25 ± 0.01 g. The testing profile began at a temperature of 50 °C and 960 RPM, which then decreased to 160 RPM after 10 s.

#### Total phenolic content (TPC)

The filtered extract (100 µL) of dry uncooked pasta was combined with distilled water (900 µL) and Folin-Ciocalteu reagent (100 µL). After 5 min, 300 µL of a 20% sodium carbonate solution was added to the mixture, which was then incubated in the dark for 1 h at room temperature. The absorbance was then measured with a spectrophotometer at a wavelength of 765 nm. The total phenolic content (TPC) was quantified (mg GAE/g sample) via the modified approach described by^35^.

#### Color instrumental analysis

The pasta samples were cooked to the optimum cooking time for each sample. The samples were placed in Petri dishes so that they evenly covered the entire surface of the dish. The samples were left to cool for ten minutes to room temperature. Excess moisture was gently removed using filter paper. Measurements were taken at three different points on the surface via a Chroma Meter (Model: CR-400, Minolta, Japan), a tri-stimulus colorimeter featuring a contact surface diameter (0.8 cm). Calibration was conducted using a white color standard before the measurements to ensure precision. The color parameters were determined according to the CIElab color system, which includes L*, a*, and b* values: L* represents brightness on a scale from 0 (dark) to 100 (light), a* indicates greenness to redness ranging from green (-a*) to red (+ a*), and b* denotes blueness to yellowness from blue (-b*) to yellow (+ b*), as described by^36^.

#### Protein digestibility

The approach outlined by^37^ was employed to analyze the in vitro digestion of proteins. In summary, flour samples were dissolved in distilled water to reach a concentration of 6.25 mg of protein per mL, and the pH was adjusted to 8.0. Following an incubation period at 37 °C, trypsin was added at a concentration of 1.6 mg/mL, and the change in pH was recorded after 10 min. The protein digestibility percentage was calculated via the following formula: % = 210.46 − 18.10 · pH.

#### Sensory evaluation of gluten-free pasta

Sensory evaluation was conducted on freshly cooked pasta samples. After cooking to their optimum times, the samples were drained and served warm at 40 ± 2 °C. A trained panel of 20 members from the Food Technology Department at the National Research Centre performed the evaluation. Prior to the assessment, panelists underwent orientation sessions following the procedure described by^29^, during which they were trained to identify and consistently score key sensory attributes of pasta using a nine-point hedonic scale. The evaluated attributes included color, taste, texture, flavor, and overall acceptability. The nine-point scale was verbally anchored with the following categories: like extremely, like very much, like moderately, like slightly, neither like nor dislike, dislike slightly, dislike moderately, dislike very much, and dislike extremely. The quality attributes of each gluten-free pasta formulation were assessed in comparison to a control sample made from 100% rice flour.

### Statistical analysis

The data were statistically analyzed via one-way analysis of variance (ANOVA) via the Statistical Analysis System software. Differences among the mean values were compared via Duncan’s multiple range test at a significance level of 95% (*P* ≤ 0.05). The results followed by different alphabetical letters significantly differ. A three-dimensional contour plot was used as a method to study the Sigma Plot Program, with the response surface for the hardness of dry rice pasta (Y) as the dependent variable and the xanthan gum (XG) concentration and guar gum (GG) concentration (X and Z) as the independent variables. The response surface methods were applied via the Sigma plot program to locate the optimum XG and GG concentrations to obtain the optimum hardness parameter that can produce high-quality rice pasta. The model proposed for the three-dimensional response surface of Y is as follows:

1 $$Y=y_\circ+ax+bz+cx^2+dz^2+ex^3+gz^3+hx^4+iz^4+jx^2z^2+kx^3z^3+lx^4z^4$$

## Results and discussion

### Approximate analysis of Raw materials

The chemical compositions of the raw materials, including rice flour (RF), oat flour (OAF), and lentil flour (LF), are presented in Table 3, highlighting the significant differences in their nutritional profiles. Rice flour (RF) has a moisture content of 7.67% and contains the lowest amounts of protein (8.15%) and ash (0.61%), but it has a high NFE value of 89.83%, indicating that it is primarily composed of carbohydrates. These findings align with those of^38^, who reported similar values for protein (7.16%), ash (0.57%), and NFE (89.56%) in rice flour, although they noted higher levels of fat (1.50%) and crude fiber (1.21%).

**Table 3:** Chemical composition of the Raw materials (mean values ± SD).

Samples	Moisture	Approximate analysis (g/100 g on dwb)				
		Ash	Protein	Lipids	CF	NFE
Rice flour	7.67^b^ ± 0.15	0.61^c^ ± 0.02	8.15^c^ ± 0.05	0.68^b^ ± 0.04	0.75^c^ ± 0.02	89.83^a^ ± 0.01
Oat flour	10.26^a^ ± 0.04	1.47^b^ ± 0.02	13.68^b^ ± 0.03	4.88^a^ ± 0.14	6.28^a^ ± 0.15	73.70^b^ ± 0.34
Lentil flour	6.03^c^ ± 0.08	2.86^a^ ± 0.04	26.09^a^ ± 0.06	0.71^b^ ± 0.02	3.44^b^ ± 0.04	66.92^c^ ± 0.04
LSD at 0.05	0.208	0.177	0.127	0.183	0.196	0.982

CF, Crude fiber; NFE, nitrogen-free extract.

Mean (*n* = 3) with different letters in the same column indicate significant differences (*P* < 0.05).

In contrast, oat flour (OAF) significantly showed the highest moisture content at 10.26% and has moderate concentrations of protein (13.68%) and ash content (1.47%). OAF also contained the highest values of lipids (4.88%) and crude fiber (6.28%) with significant differences compared to other samples, indicating a more nutritionally balanced profile. These findings align with those of^39^, who reported that oats contain approximately 60% starch, 7% lipids, and 14% protein. Additionally^40^, reported that the protein content of oat grains typically ranges from 11 to 15% on a dry weight basis.

Our results show that LF significantly recorded the lowest value of moisture content (6.03%) and the highest protein content (26.09%) with 2.86% of ash content, highlighting its nutritional density, particularly in terms of protein. It also contains lower lipid levels (0.71%) and an NFE of 66.92%, indicating a significantly lower carbohydrate content, although this is lower than that in RF or OAF. Overall, these findings indicate that LF is the most protein-rich, OAF is rich in fiber and lipids, and RF is predominantly carbohydrate-based among the three types. The chemical composition of lentil seeds aligns well with the criteria for an “ideal diet,” as they have a low-fat content (approximately 0.7–3.4%) and a substantial amount of high-quality protein (ranging from 21 to 29%)^11^.

### Mineral contents of the Raw materials

The mineral profiles of RF, OAF, and LF significantly differ in their essential mineral contents, as presented in Table 4. Rice flour significantly (*P* < 0.05) showed the lowest values of iron, zinc, potassium, and magnesium, indicating a relatively low mineral density compared with those of the other flours. In contrast, oat flour contains relatively high levels of iron (1.71 mg/100 g), zinc (0.90 mg/100 g), potassium (303.30 mg/100 g), and magnesium (107.47 mg/100 g) compared to RF with significant differences, making it a more substantial source of these minerals, particularly potassium and magnesium. These results align with findings of^41^, who reported a zinc content in oats ranging from 0.61 to 6.86 mg/100 g. However, they noted a greater range of iron (3.47–10.38 mg/100 g), which suggests that the reduction in iron levels may be due to challenges in milling whole oats, potentially diminishing their mineral content.

**Table 4:** Mineral composition of the Raw materials (mean values ± SD).

Samples	Minerals content (mg/100 g on dry weight basis)			
	Iron	Zinc	Potassium	Magnesium
Rice flour	0.69^c^ ± 0.02	0.58^c^ ± 0.06	101.00^c^ ± 4.24	34.61^c^ ± 2.15
Oat flour	1.71^b^ ± 0.06	0.90^b^ ± 0.03	303.30^b^ ± 6.22	107.47^b^ ± 5.76
Lentil flour	4.68^a^ ± 0.04	2.65^a^ ± 0.04	771.00^a^ ± 15.56	146.50^a^ ± 3.54
LSD at 0.05	0.085	0.108	19.868	7.620

Mean (*n* = 3) with different letters in the same column indicate significant differences (*P* < 0.05).

Other studies have reported similar ranges for iron (2.50-3.00 mg/100 g), zinc (1.60-2.00 mg/100 g), potassium (241.70-258.30 mg/100 g), and magnesium (62.40–89.10 mg/100 g)^40^, which aligns with the current findings. The variations observed may be due to genetic differences and agronomic practices during production^28^. Notably, LF stands out for its superior mineral content, with the highest levels of iron (4.68 mg/100 g), zinc (2.65 mg/100 g), potassium (771.00 mg/100 g), and magnesium (146.50 mg/100 g) with significant differences compared to RF and OAF. This makes LF the more mineral-rich option, increasing the intake of essential minerals such as iron, zinc, and potassium.

These variations highlight the potential of LF as a valuable mineral source in dietary applications. A study by^42^ on raw lentils revealed that they contained 7.30 mg/100 g of iron, 4.30 mg/100 g of zinc, 960 mg/100 g of potassium, and 138 mg/100 g of magnesium. The differences in mineral content may be due to genetic factors and agronomic practices during production. Furthermore^43^, reported that the genotype and soil mineral composition significantly affect the mineral content of lentils.

### Pasting behavior of the prepared formulas

Pasting properties are crucial indicators of pasta quality^44,45^ reported that these properties impact the cooking qualities and sensory properties of rice noodles. In the food industry, pasting parameters are important for determining the textural quality of the final product^45,46^. As illustrated in Table 5, differences exist in the pasting parameters among the formulations. C1, made entirely from RF, significantly (P 0.05) presented the highest peak viscosity (PV) at 684.10 cP, indicating strong pasting capabilities. in contrast to the LF that significantly showed the lowest PV value (60.68 cP). Also, C2 consisting solely of OAF, had a lower PV (488.00 cP) compared to C1, suggesting a weaker pasting performance. The peak viscosity is the maximum viscosity of gelatinized starch during heating in water and reflects the water-binding capacity of starch granules^47^.

**Table 5:** Pasting behavior.

Pasting parameters	LF	Prepared formulas						LSD at 0.05
		Control samples		Mixture samples				
		C1	C2	M1	M2	M3	M4	
PV (cP)	60.68 g ± 2.45	684.10a ± 12.46	488.00b ± 8.87	444.70c ± 10.40	391.10d ± 5.94	343.30e ± 11.55	302.60f ± 7.09	16.987
T (min)	14.90a ± 0.03	13.10b ± 0.12	12.00c ± 0.08	11.50d ± 0.10	11.30e ± 0.05	11.40e ± 0.18	13.10b ± 0.01	0.242
PT (°C)	61.80e ± 0.56	59.10f ± 0.32	63.40d ± 0.22	59.80f ± 0.09	72.20a ± 0.60	69.90b ± 0.27	68.10c ± 0.59	0.829
Peak T. (°C)	94.80a ± 0.91	95.00a ± 1.03	94.90a ± 0.88	94.90a ± 0.43	94.90a ± 1.12	94.90a ± 0.64	95.00a ± 0.31	1.683
HS (cP)	59.46 g ± 2.86	548.90a ± 4.61	357.60c ± 2.39	395.70b ± 1.17	351.20d ± 1.80	318.30e ± 2.88	292.70f ± 2.60	5.044
BD (cP)	1.21 g ± 0.02	99.18b ± 1.03	130.40a ± 1.75	48.96c ± 1.22	39.92d ± 1.37	25.16e ± 0.60	9.94f ± 0.28	3.323
FV (cP)	89.88f ± 1.10	1093.00a ± 15.18	928.60b ± 17.27	925.80b ± 8.93	830.00c ± 11.35	743.20d ± 9.20	682.20e ± 10.39	22.842
ST(cP)	88.67f ± 2.81	993.60a ± 16.72	798.20c ± 19.67	876.8b ± 8.52	790.10c ± 14.25	718.00d ± 12.16	672.20e ± 13.28	20.470

PV, peak viscosity; T, peak time; PT, pasting temperature; Peak T, peak temperature; HS, holding strength; BD, breakdown; FV, final viscosity; ST, setback from the trough; LF, lentil flour; C1, 100% rice flour; C2, 100% oat flour; M1, 50% rice flour + 50% oat flour, M2 = 45% rice flour + 45% oat flour + 10% lentil flour; M3, 40% rice flour + 40% oat flour + 20% lentil flour; M4, 35% rice flour + 35% oat flour + 30% lentil flour.

Mean (*n* = 2) with different letters in the same column indicate significant differences (*P* < 0.05).

The peak time (T) showed relative uniformity across the formulas, with values between 11.30 and 14.90 min. Concerning the pasting temperature (PT), the oat flour (C2) significantly recorded a higher value (63.40 °C) compared to rice flour (C1: 59.10 °C) and lentil flour (LF: 61.80 °C), indicating that OAF needs more heat for gelatinization. The peak temperature (Peak T) ranged from 94.90 °C to 95.00 °C, where this variable indicated the influence of various formulas on the gelatinization temperature, where there were no significant differences between all samples regarding this parameter. With respect to the holding strength (HS), C1 had the highest HS at 548.90 cP, on the contrary, the lowest value (59.46 cP) significantly recorded by LF. Consequently, HS values were significantly decreased in all the mixed formulations with the increasing level of LF, recording the lowest HS value (292.70 cP) with M4, reflecting reduced viscosity stability during the cooling phase.

Likewise, the breakdown (BD) viscosity, which measures how stable the paste is under shear stress^48^, decreased as more LF was added. Where the paste reached the lowest degree of stability in M4, which had a lower breakdown at 9.94 cP. The breakdown occurred due to the structural disruption of gelatinized starches at elevated temperatures, which was influenced by the amylose content and the fine structure of amylopectin^49^. The highest value of final viscosity (FV, the viscosity of the sample at the end of the test at 50 °C) was significantly recorded by C1 (1093.00 cP), compared to the lowest value (89.88 cP) that was obtained by LF, where the FV progressively reduced in the mixed formulations with the increasing level of LF, to record 682.20 cP with M4 sample.

The setback from trough (ST) followed a similar FV pattern, where the LF significantly recorded the lowest ST value (88.67 cP), therefore, the LF had a significant role in reducing the final viscosity compared to M1, indicating diminished gel strength postcooling.

PV showed a positive correlation with amylose content, suggesting that higher amylose levels may contribute to greater initial swelling of starch granules. In contrast, BD did not exhibit a consistent correlation with either amylose content or final viscosity, likely due to variations in starch structure and botanical origin among the different formulations^50^. The breakdown value serves as an indicator of the stability of starch grains; thus, reduced values correspond to enhanced thermal stability of the paste, along with improved resistance to shear and agitation^51^.

These findings suggest that an increase in LF content generally leads to reductions in peak and final viscosities, breakdown, and setback, indicating that LF may positively affect the gel strength and stability of mixtures. The setback is a critical parameter that indicates the retrogradation propensity of a starch-based product, serving as a measure of its short-term aging capacity and cold paste stability^52^. Thus, the low propensity for short-term retrogradation in the paste may be attributed to higher protein content, where protein-rich formulas have been reported to decrease breakdown during pasting due to their interaction with starch. Protein-rich formulations have been reported to reduce breakdown during pasting due to protein-starch interactions. Upon heating, proteins may denature and form networks that restrict starch granule swelling and protect against shear-induced disintegration^53^. Consequently, samples with higher protein content, such as those containing legumes, often exhibit lower breakdown values^54–56^ also noted that increased protein content is typically associated with reduced starch concentration and breakdown. Moreover, proteins can inhibit starch retrogradation by promoting amylose-protein interactions, thereby limiting amylose-amylose reassociation^57^.

SB is influenced by the concentration and molecular weight of amylopectin within a pure starch matrix^58^. In more intricate flour systems, SB may be affected by various factors, including the starch content, the ratio of amylose to amylopectin, the structural properties of amylose, and the presence of proteins and lipids. Consequently, the viscosity values of a given material are not directly comparable to other findings (as values), even for the same material, owing to the variability introduced by factors such as the type of measuring device, the specific characteristics (particle size, amylose to amylopectin, varieties, etc.) of the material, and the ratio of water to dry matter.

The peak, trough, and final viscosities are influenced by various factors, including the morphology of starch particles, the ratio of amylose to amylopectin, the relative molecular weight, and the size of the starch particles^59^. However, relative comparisons can still be made. In general, the results of this study are consistent with those of^60^, who studied the pasting behavior of rice varieties that differed in their protein contents and reported that rice varieties with high protein contents presented low values ​​for peak viscosity, final viscosity, and setback.

### Functional properties of the prepared formulas

Table 6 lists the functional characteristics of various flour blends, with a focus on the WHC, OHC, and swelling power (SP). The findings revealed notable variations among the samples concerning each functional parameter (*p* < 0.05). Specifically, the WHC values ranged from 1.19 g/g in sample C2 (composed entirely of OAF) to 1.58 g/g in M4 (a blend of 35% RF, 35% OAF, and 30% LF), with the highest WHC associated with greater inclusion of LF that recorded the highest value of WHC (1.62 g/g). According to the statistical data, there were no significant differences (*P* ≤ 0.05) between the WHC values of RF (C1, 1.31 g/g) and the other samples containing 50% RF with 50% OAF (M1, 1.29 g/g).

**Table 6:** Functional properties of the prepared formulas.

Samples	Functional parameters		
	WHC (g/g)	OHC (g/g)	SP (g/g)
LF	1.62^a^ ± 0.04	1.86^a^ ± 0.02	6.59^b^ ± 0.03
C1	1.31^d^ ± 0.03	1.57^d^ ± 0.02	6.88^a^ ± 0.11
C2	1.19^e^ ± 0.01	1.80^ab^ ± 0.04	4.96^d^ ± 0.09
M1	1.29^d^ ± 0.02	1.71^c^ ± 0.01	6.22^c^ ± 0.02
M2	1.41^c^ ± 0.01	1.75^bc^ ± 0.01	6.29^c^ ± 0.04
M3	1.51^b^ ± 0.01	1.79^ab^ ± 0.01	6.46^bc^ ± 0.03
M4	1.58^a^ ± 0.01	1.83^a^ ± 0.01	6.56^b^ ± 0.01
LSD at 0.05	**0.067**	**0.076**	**0.251**

Mean (*n* = 3) in the same column with different letters are significantly different (*p* < 0.05).

WHC, water holding capacity; OHC, oil holding capacity; SP, swelling power; LF, lentil flour; C1, 100% rice flour; C2, 100% oat flour; M1, 50% rice flour + 50% oat flour; M2, 45% rice flour + 45% oat flour + 10% lentil flour; M3, 40% rice flour + 40% oat flour + 20% lentil flour; M4, 35% rice flour + 35% oat flour + 30% lentil flour.

In terms of the OHC, RF significantly showed the lowest value (1.57 g/g), whereas LF and OAF presented a relatively high OHC value (1.86 and 1.80 g/g, respectively), while the blended formula (M1: 50% RF + 50% OAF) recorded 1.71 g/g of OHC. Data also indicated that LF caused a significant increase in OHC compared to M1 when replaced by 20% and 30% of LF (M3: 1.79 and M4: 1.83, respectively). With regard to the swelling power (SP), the maximum values (6.88 and 6.59 g/g) were observed by C1 and LF, respectively. Conversely, the lowest SP value (4.96 g/g) significantly observed by C2 (100% OAF). On the other side, the SP values were gradually increasing with the increasing level of LF. This may be due to the higher protein content and most likely due to the lower amylose content, which plays a key role in swelling^61^. The results of the current study are also consistent with those of^62^, who reported that higher protein content of composite blends leads to increased water absorption and swelling capacity.

The progressive addition of LF from M2 to M4 generally increased both the WHC and OHC, although it resulted in a slight decrease in SP compared with RF (C1). These findings imply that the inclusion of LF improves the water and oil absorption properties of flour blends, which could influence the texture and mouthfeel of food products, albeit at the expense of their swelling capacity.

Although the peak viscosity (PV) is related to water binding, the sample containing 100% RF (C1), which presented high viscosity (684.10 cP, Table 5), presented a lower WHC value than did the samples containing different proportions of LF, as these results are not consistent with the hypothesis that high PV is accompanied by high WHC^63^. The gradual increase in WHC, which is proportional to the increase in the proportion of LF, may be due to the ability of LF to bind water at low temperatures compared with RF, which absorbs water at high temperatures. This can be observed through the ability of RF to swell at higher temperatures than those of the other samples. Starch granules typically exhibit insolubility in water at temperatures below 50 °C^64^.

Nevertheless, upon reaching a specific temperature threshold, these granules demonstrate significant water uptake and concurrently undergo a volumetric expansion that can be several times greater than their original size, leading to a marked increase in viscosity^65^. Moreover, the increase in WHC is most likely due to the high protein content and starch structure^66^, which in turn is consistent with the findings of^67^, who reported that high-protein rice has the potential to increase the WHC and impede the movement of water during the retrogradation process of rice starch gels. Additionally, the increase in the OAC of LF might be due to the increase in lipophilic amino acids^68^.

### Optimization of gluten-free pasta hardness via Xanthan gum and Guar gum: A response surface methodology approach

**Fig. 1:**
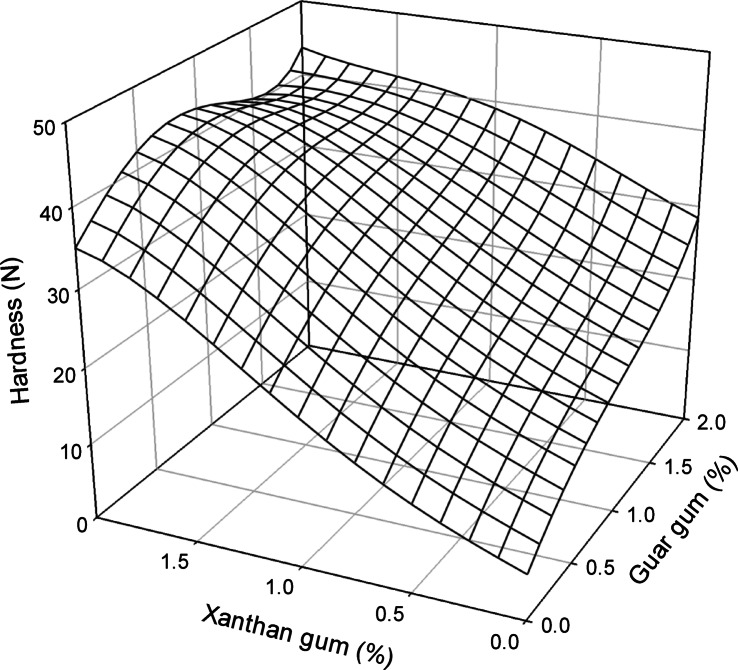
Three-dimensional regression plot to predict the hardness parameter against different XG and GG levels.

Three-dimensional response surface analysis was essential for determining the optimal combined levels of XG and GG. Figure 1 shows the response surface, which shows the hardness properties of dry pasta with different XG and GG concentrations. The regression coefficients corresponding to the various levels of XG and GG that affect the pasta’s hardness are also shown in Table 7.

**Table 7:** Regression coefficient response of different concentrations of XG and GG to dry pasta hardness.

Variables	Hardness (*N*)
Linear	6.0253
intercept	
a	16.1642
b	8.2940
Quadratic	
c	-5.2128
d	4.8474
Cubic	
e	-0.9439
g	1.9429
Quaternary	
h	1.1528
i	-1.3589
Quadratic interaction	
j	1.1275
Cubic interaction	
k	-1.4640
L	0.2390
r^2^	0.9468

**r**^**2**^ = Determination coefficient.

The impact of XG and GG concentrations on the hardness of gluten-free rice pasta is assessed in this study using response surface methodology (RSM). Several important insights are obtained from the regression analysis and the given equation. These coefficients show how each term-linear, quadratic, and cubic-affects the pasta’s hardness.

The direct effects of XG and GG on the pasta’s hardness are shown by the linear coefficients (a and b, 16.1642 and 8.2940, respectively). With negative quadratic coefficients (c and e) for XG, the quadratic and cubic terms show the curvature and nonlinear effects, indicating that a drop in hardness could result from an increase in its concentration above a certain threshold. Conversely, the positive coefficients for GG (d and g) imply that hardness tends to increase with increasing concentration up to a certain limit. The interaction terms (j, k) and the quaternary terms (h, i, L) illustrate the combined effects of XG and GG, emphasizing the intricate nature of their relationship in achieving optimal pasta hardness.

The model’s dependability is confirmed by its strong fit, as evidenced by its R^2^ value of 0.9468, which shows that the concentrations of XG and GG account for 94.68% of the variation in pasta hardness. The ideal XG and GG concentrations for reaching the required hardness were determined based on the analysis and 3D regression diagram (Fig. 1). The optimal hardness value for the combination of XG and GG across various levels was determined to be 44 N. The quaternary model (Eq. 1) demonstrated the most effective relationship between the levels of XG and GG for achieving the maximum hardness value (r² = 0.9468), represented by the following equation:

$$\begin{gathered} {\text{Y}} = {\text{6}}.0{\text{253}} + {\text{16}}.{\text{1642X}} + {\text{8}}.{\text{294}}0{\text{Z}} - {\text{5}}.{\text{2128X}}^{{\text{2}}} + {\text{4}}.{\text{8474Z}}^{{\text{2}}} - 0.{\text{9439X}}^{{\text{3}}} + {\text{1}}.{\text{9429Z}}^{{\text{3}}} + {\text{1}}.{\text{1528X}}^{{\text{4}}} - {\text{1}}.{\text{3589Z}}^{{\text{4}}} \hfill \\ \quad \;\; + {\text{ 1}}.{\text{1275X}}^{{\text{2}}} {\text{Z}}^{{\text{2}}} - {\text{1}}.{\text{464}}0{\text{X}}^{{\text{3}}} {\text{Z}}^{{\text{3}}} + 0.{\text{239}}0{\text{X}}^{{\text{4}}} {\text{Z}}^{{\text{4}}} \hfill \\ \end{gathered}$$

Figure 1 was instrumental in predicting the ideal levels of XG and GG. Plotting the XG and GG levels allowed for the response surface analysis to get this data. High-quality gluten-free pasta can be made using the ideal concentrations of XG and GG, which were found to be 0.75% and 2.00%, respectively.

The nonlinear and intricate link between gum concentrations and pasta hardness is reflected in the model’s inclusion of quadratic and cubic factors. For example, the negative quadratic term for XG (X²) indicates that a decrease in hardness occurs when its concentration is increased above a particular threshold. This is probably because of excessive water binding and gel formation, which weakens the dried pasta’s internal structure. On the other hand, GG’s positive quadratic and cubic terms show a steady rise in hardness up to a concentration where the gum best supports the matrix. These nonlinear patterns correlate to physical changes that are known to affect the texture and resilience of gluten-free pasta, including reduced porosity, enhanced film development around starch granules, and increased matrix cohesion.

The ideal concentrations of XG (0.75%) and GG (2.00%), as indicated by the RSM, were used in a confirmation experiment to validate the model. There was a residual of − 2.52 N since the observed hardness was 41.48 N, but the expected value was 44.00 N. This minor variation validates the model’s applicability in actual formulation circumstances and supports its predictive adequacy.

### Hardness of uncooked pasta

**Fig. 2:**
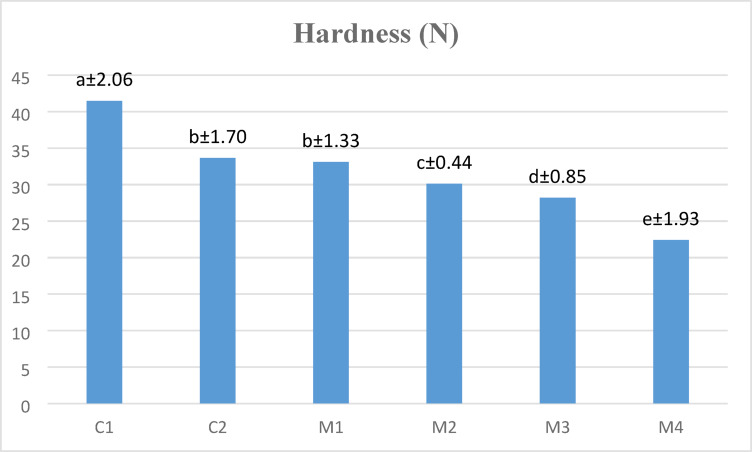
Hardness values of uncooked pasta samples. Means (*n* = 3) ± SD with different small letters are significantly different (*P* < 0.05).

The hardness measurements of the raw pasta samples are displayed in Fig. 2, which indicates significant variations that are ascribed to the flour content. With the greatest hardness value of 41.48 N, the rice pasta (C1)—which is made entirely of RF—showed a harder texture than the other samples. This finding is corroborated by the data in Fig. 2, where the RF-based blend (C1) presented the highest peak viscosity, which is generally associated with firmer pastes and, consequently, firmer pasta products. In contrast, the oat pasta (C2), made solely from OAF, presented a lower hardness value of 33.64 N, which corresponds with its lower peak viscosity (PV) and elevated pasting temperature (Table 5).

As LF was integrated into the rice-oat blends (M2 to M4), a progressive reduction in hardness values was observed. For example, M1 (50% RF + 50% OAF) presented a hardness of 33.09 N, whereas M4 (35% RF + 35% OAF + 30% LF) presented the lowest hardness at 22.40 N. This decline in hardness aligns with the reductions in peak viscosity, final viscosity, and holding strength (HS) documented in Table 5 for blends with increasing LF contents. Due to RF containing a higher protein, fiber, and water-binding ability, pasta hardness gradually decreases as the amount of LF increases. Dietary fiber causes increased water absorption during dough formation, which prevents a hard gel network from forming. Furthermore, by reducing starch-protein interactions, the higher protein concentration may prevent starch from gelatinizing, making the pasta structure softer and less cohesive. In conclusion, the patterns in pasta hardness that have been found correspond to the findings of pasting behavior; a higher percentage of RF produces firmer pasta and higher pasting viscosity, whereas a higher percentage of LF produces softer pastes and samples of uncooked pasta.

The increased hardness of raw pasta made from 100% rice flour (C1) can be explained by its elevated peak viscosity (Table 5), which contributes to enhanced cohesion. According to^14^, the hardness values of cookies made with LF were significantly lower (*p* < 0.05) than those of the control cookies (100% wheat flour)^69^. attributed the decrease in the hardness value of cookies to the formation of a weaker cookie matrix due to the structural degradation of starch and protein.

### Approximate analysis of cooked pasta

Table 8 shows the chemical compositions of various cooked pasta samples, revealing notable differences among the different formulations. The moisture content varied, with rice pasta (C1) accounting for 11.13% and oat pasta (C2) accounting for 12.19%, indicating that oat pasta retains slightly more moisture than its rice counterpart. The ash content of C2 was greater than that of rice pasta at 1.49%, implying a greater mineral content, with significant differences between them (*P* ≤ 0.05), with an ash content of 0.66%. The protein content was also greater in the oat pasta (13.02%), significantly exceeding that in the rice pasta (7.88%). Oats are inherently rich in various health-enhancing constituents, including proteins, fibers, and minerals^70^.

**Table 8:** Chemical composition of cooked pasta samples (means ± SD).

Pasta samples	Moisture	Approximate analysis (g/100 g on dwb)				
		Ash	Protein	Lipids	CF	NFE
C1	11.13^c^ ± 0.14	0.66^e^ ± 0.01	7.88^f^ ± 0.08	0.59^e^ ± 0.02	0.69^d^ ± 0.01	90.19^a^ ± 0.06
C2	12.19^a^ ± 0.13	1.49^b^ ± 0.02	13.02^c^ ± 0.09	4.79^a^ ± 0.04	6.57^a^ ± 0.05	74.15^f^ ± 0.20
M1	11.79^ab^ ± 0.02	1.06^d^ ± 0.01	10.20^e^ ± 0.02	2.61^b^ ± 0.05	3.73^b^ ± 0.01	82.41^b^ ± 0.07
M2	11.70^ab^ ± 0.19	1.27^c^ ± 0.03	12.11^d^ ± 0.03	2.48^b^ ± 0.03	3.58^b^ ± 0.04	80.56^c^ ± 0.01
M3	11.71^ab^ ± 0.15	1.46^b^ ± 0.03	13.61^b^ ± 0.02	2.30^c^ ± 0.02	3.61^b^ ± 0.05	79.04^d^ ± 0.04
M4	11.62^bc^ ± 0.12	1.65^a^ ± 0.02	15.12^a^ ± 0.04	2.10^d^ ± 0.04	2.66^c^ ± 0.04	78.49^e^ ± 0.13
LSD at 0.05	0.542	0.090	0.213	0.132	0.151	0.418

Means (*n* = 3) in the same column with different letters are significantly different (*p* < 0.05).

C1, rice paste (100% rice flour); C2, oat paste (100% oat flour); M1, treatment 1 (50% rice flour + 50% oat flour); M2, treatment 2 (45% rice flour + 45% oat flour + 10% lentil flour); M3, treatment 3 (40% rice flour + 40% oat flour + 20% lentil flour); M4, treatment 4 (35% rice flour + 35% oat flour + 30% lentil flour); CF, crude fiber; NFE, nitrogen-free extract.

Research indicates that OAF has a significantly greater protein content than wheat, rice, maize, rye, barley, and sorghum, which have been increasingly employed in the fortification of food products because of their exceptional nutritional profile, particularly their elevated levels of dietary fiber, vitamins, and minerals^71^. As demonstrated by formulation M4 (35% RF + 35% OAF + 30% LF), which had the greatest protein concentration at 15.12%, this trend continued with the inclusion of LF. Rice pasta had the lowest lipid level (0.59%), while C2 had the highest lipid content (4.79%). Crude fiber (CF) and NFE displayed a similar pattern, with oat pasta and lentil-enriched formulations demonstrating higher fiber levels and lower NFE values than those of rice pasta.

Notably, M4, which contained the highest proportion of LF, presented the lowest NFE at 78.49% for mixture and a moderate fat content of 2.10%. These results imply that LF reduces total NFE in addition to raising protein levels. According to these findings, adding OAF and LF to pasta recipes improves their nutritional profile by raising their protein, fiber, and mineral contents, making them more nutrient-dense than rice-based pastas. Additionally, LF helps create a more balanced chemical composition with higher levels of fiber and protein and lower levels of carbohydrates. Following the addition of LF^72^, observed a rise in ash, fiber, and protein levels along with a decrease in moisture and fat content.

Furthermore^73^, found that adding oats and LF to wheat biscuits improved the product’s nutritional profile by increasing its protein and ash content while lowering its total carbohydrate content. The nutritional advantages of legumes as high-protein ingredients in baked products and noodles have been the subject of numerous research studies^74–76^.

### Mineral content of cooked pasta

The mineral compositions of pasta samples are displayed in Table 9, which also demonstrates that the type of flour used significantly affects the quantities of iron, zinc, potassium, and magnesium. The lowest mineral levels were found in rice pasta (C1), which was made entirely of RF. Its components included iron (0.51 mg/100 g), zinc (0.39 mg/100 g), potassium (83.88 mg/100 g), and magnesium (27.64 mg/100 g) (*P* ≤ 0.05). In contrast, oat pasta (C2), made from 100% OAF, presented significantly elevated mineral levels compared with those of C1, which was likely attributable to the inherently higher mineral content found in oats. The introduction of LF into the rice-oat pasta blends (M2 to M4) resulted in a pronounced increase in all the mineral concentrations.

**Table 9:** Mineral contents of the prepared pasta samples.

Pasta samples	Minerals content (mg/100 g on dwb)			
	Iron	Zinc	Potassium	Magnesium
C1	0.51^e^ ± 0.03	0.39^d^ ± 0.02	83.88^e^ ± 3.59	27.64^e^ ± 2.84
C2	1.37^c^ ± 0.08	0.62^c^ ± 0.04	266.70^c^ ± 6.62	89.12^b^ ± 1.80
M1	1.03^d^ ± 0.06	0.59^c^ ± 0.03	186.90^d^ ± 7.81	57.87^d^ ± 3.32
M2	1.36^c^ ± 0.06	0.77^b^ ± 0.04	259.23^c^ ± 6.22	69.80^c^ ± 2.15
M3	1.70^b^ ± 0.11	0.93^b^ ± 0.06	316.38^b^ ± 8.43	87.55^b^ ± 5.09
M4	2.03^a^ ± 0.13	1.13^a^ ± 0.06	377.01^a^ ± 7.90	104.61^a^ ± 3.47
LSD at 0.05	0.203	0.172	12.875	7.889

Means (*n* = 3) in the same column with different letters are significantly different (*p* < 0.05).

C1, rice paste (100% rice flour); C2, oat paste (100% oat flour); M1, treatment 1 (50% rice flour + 50% oat flour); M2, treatment 2 (45% rice flour + 45% oat flour + 10% lentil flour); M3, treatment 3 (40% rice flour + 40% oat flour + 20% lentil flour); M4, treatment 4 (35% rice flour + 35% oat flour + 30% lentil flour).

In particular, the highest levels of iron (2.03 mg/100 g), zinc (1.13 mg/100 g), potassium (377.01 mg/100 g), and magnesium (104.61 mg/100 g) were found in the M4 pasta samples (35% RF, 35% OAF, and 30% LF). This suggests that the addition of LF significantly increased the mineral profile. These findings suggest that adding LF to pasta recipes not only raises the amounts of protein and fiber but also significantly improves the amounts of minerals, especially iron, zinc, potassium, and magnesium. As a result, these pasta types are more nutritious than those made only with RF or OAF. Others have shown that, when compared to the control sample (100% wheat flour), the addition of oat and LF significantly improves the final product’s mineral composition, especially in terms of iron and zinc levels^73,77^.

### Protein digestibility

**Fig. 3:**
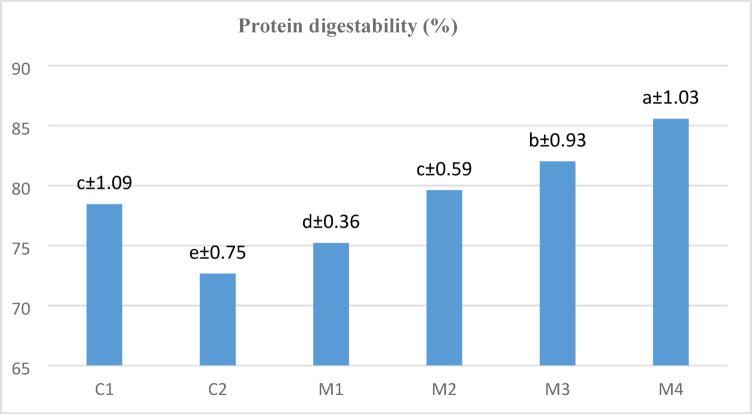
Protein digestibility of cooked pasta. Means (*n* = 3) ± SD with different small letters are significantly different (*P* < 0.05).

Evaluating agricultural proteins’ digestibility provides important information for food formulation procedures. This analysis suggests that crop proteins can be used as single or combined ingredients to enhance flavor, aid in digestion, and boost nutritional content^78^. The cooked pasta samples’ protein digestibility values, as shown in Fig. 3, highlight how different flour compositions affect protein bioavailability.

The protein digestibility of the rice pasta (C1), which was manufactured totally of RF, was 78.45%, much higher than that of the oat pasta (C2), which was made entirely of OAF, with a digestion of 72.66%. Protein digestibility increased gradually when LF was introduced to the rice-oat pasta mixes (M2 to M4); the maximum digestibility (85.57%) was obtained with M4 (35% RF + 35% OAF + 30% LF) with significant differences compared to other samples. This improvement in digestibility is probably due to LF’s higher-quality protein and advantageous amino acid profile, which enhance the pasta formulation’s overall digestibility. Additionally, the protein digestibility of the M3 pasta sample (40% RF + 40% OAF + 20% LF) significantly increased to 82.01% followed by M2 (79.61%). While the low protein digestibility of raw lentils is mostly caused by the presence of antinutrients^79^, pasta’s protein digestibility is improved by cooking since the cooking process efficiently reduces the antinutrient levels in the finished product.

The hydrolysis of indigestible proteins, the inactivation of protease inhibitors, and the increase in protein solubility all have a significant impact on the increase in protein digestibility of LF, according to^80^. Extrusion, cooking, and baking are some of the food processing methods that can change the secondary structure of proteins in LF. Rapid protein hydrolysis is encouraged by these alterations, which make it easier for digestive enzymes to reach peptide bonds^81^.

These findings suggest that adding LF to rice-oat pasta mixes greatly improves protein digestibility, which may make these mixtures more nutritionally beneficial than regular rice or oat pasta. Therefore, legume protein may be a suitable ingredient for athletes looking for easily digestible, high-protein foods to improve their performance, and adding lentil proteins to brown rice noodles has been demonstrated to reduce the glycaemic index^82^. Finally, leguminous proteins showed higher digestibility rates than cereals, suggesting that leguminous proteins have a more flexible structure that permits their use in food processing and other applications^78^.

Proteins that have been thermally denaturated tend to be more digestible^83^. Both the type of protein involved and the degree of heat treatment have an impact on how much of this increase occurs. Depending on the situation, proteins may congregate or unravel from their compact conformations, increasing the amount of peptide chains available for hydrolytic enzymes. In the processing of beans, heat treatment leads to a noticeable improvement in digestibility. According to^84^, beans’ protein digestibility is greatly increased by soaking and boiling them.

### Total phenolic content

**Fig. 4:**
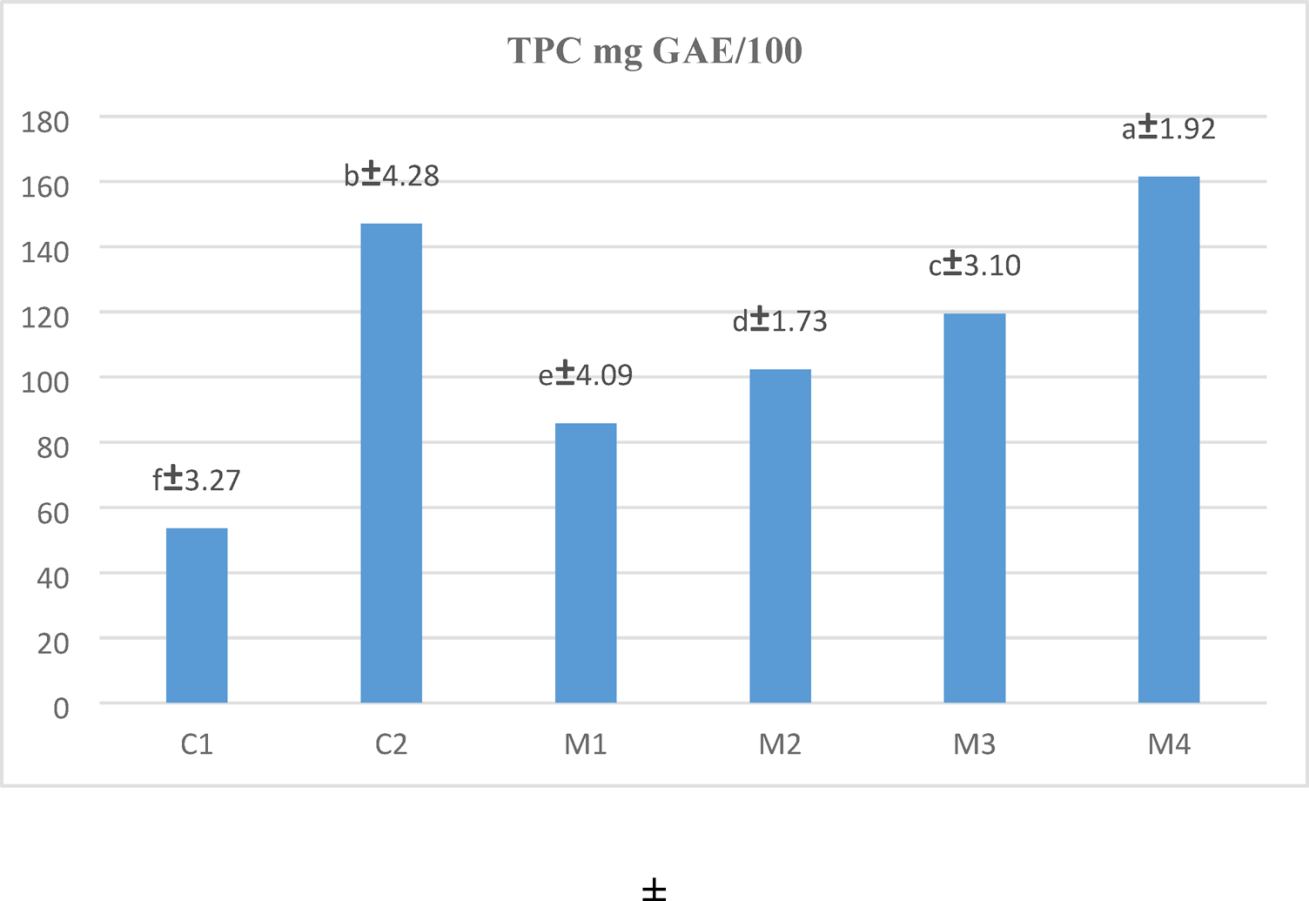
Total phenolic content of cooked pasta. Means (*n* = 3) ± SD with different small letters are significantly different (*P* < 0.05).

The total phenolic content (TPC) of the different pasta samples is presented in Fig. 4, highlighting significant differences (*P* < 0.05) among the various composite blends. Rice pasta (C1), made entirely from (RF), exhibited the lowest TPC value of 53.61 mg GAE/100 g, indicating a limited presence of polyphenols. Conversely, oat pasta (C2), composed solely of OAF, showed a significantly higher TPC of 147.01 mg GAE/100 g, reflecting the naturally higher polyphenol content in oats. The gradual incorporation of LF into the rice-oat blends (M2 to M4) led to a progressive increase in TPC. The M4 pasta sample (35% RF, 35% OAF, and 30% LF) recorded the highest TPC value at 161.45 mg GAE/100 g, demonstrating the enhancing effect of LF on the phenolic content of the pasta.

These results suggest that the addition of LF to rice-oat pasta blends contributes to an increased total phenolic content, indicating an enrichment in bioactive compounds. Phenolic compounds and certain polypeptides may contribute to antioxidant properties, according to^71,85^.

### Cooking quality of prepared pasta

Table 10 presents the cooking quality parameters of various pasta samples, revealing notable differences among the different formulations. The optimum cooking time (OCT) varied from 10.35 min for oat pasta (C2) to 17.31 min for Treatment 4 (M4), which consisted of 35% RF, 35% OAF, and 30% LF. This variation suggests that a higher percentage of LF is associated with a longer cooking time, most likely because the LF forms a more complex starch-protein matrix that may require more time for full hydration and cooking. According to the statistical data, a significant difference (*P* ≤ 0.05) was found between all samples except C1 and M2, there are no significant differences between them concerning the OCT.

**Table 10:** Cooking quality of pasta samples.

Samples	Cooking quality parameters				
	OCT min	CY (%)	CL (g/100 g)	SI	NL %
C1	13.77^c^ ± 0.23	293.50^ab^ ± 4.95	9.10^d^ ± 0.19	3.26^ab^ ± 0.08	2.78^bc^ ± 0.03
C2	10.35^e^ ± 0.21	249.00^d^ ± 8.49	11.36^a^ ± 0.11	2.68^e^ ± 0.03	4.01^a^ ± 0.18
M1	12.09^d^ ± 0.06	266.00^cd^ ± 5.66	10.41^b^ ± 0.27	2.83^de^ ± 0.04	3.11^b^ ± 0.13
M2	13.42^c^ ± 0.09	274.50^bcd^ ± 3.54	9.94^bc^ ± 0.08	2.92^cd^ ± 0.04	3.04^bc^ ± 0.05
M3	14.80^b^ ± 0.28	280.50^bc^ ± 9.90	9.61^cd^ ± 0.05	3.09^bc^ ± 0.04	2.95^bc^ ± 0.04
M4	17.31^a^ ± 0.03	308.00^a^ ± 4.24	7.80^e^ ± 0.17	3.36^a^ ± 0.08	2.72^c^ ± 0.02
LSD at 0.05	0.711	26.054	0.646	0.216	0.389

Means (*n* = 3) in the same column with different small letters are significantly different (*p* < 0.05).

OCT, optimum cooking time; CY, cooking yield; CL, cooking loss; SI, swelling index; NL, nitrogen loss.

M4 had the highest cooking yield (CY) at 308.00%, while C2 had the lowest at 249.00% followed by M1 (266.00%) with no significant differences between them. This suggests that pasta with a higher LF content absorbed more water during cooking, which led to a higher yield. Concerning the cooking loss (CL), which quantifies the amount of solids lost during cooking. M4 had the lowest loss at 7.80 g/100 g, while C2 had the most at 11.36 g/100 g with significant differences between them. This result suggests that whereas LF-enriched pasta blends show reduced losses during cooking, oat pasta tends to lose more solids. This is probably because the starch and other solids are better retained inside the cooking matrix. C2 had the lowest swelling index (SI) at 2.68, while M4 had the highest at 3.36. This implies that adding LF improves pasta’s ability to expand when cooking.

As a measure of protein stability, the nitrogen loss (NL) was estimated, and the obtained data indicated that oat pasta (C2) significantly recorded the highest value of NL (4.01%), followed by 3.11% for M1. However, the NL values were gradually decreased with the increasing level of LF until it achieved the lowest value (2.72%) for M4 with a significant difference compared to M1. This result suggests that adding LF helps reduce nitrogen loss during cooking, possibly as a result of the LF matrix’s enhanced ability to retain protein. In conclusion, while somewhat extending the cooking time, adding more LF has a good impact on a number of cooking quality metrics, such as the cooking yield, swelling index, and nitrogen retention. These changes could be related to the distinct structural and textural qualities that LF adds to the pasta matrix^86^. observed that adding LF to spaghetti increased the amount of essential amino acids, but it significantly decreased the cooking yield of cooked pasta, as evidenced by lower breaking energy and higher cooking loss.

These quality problems were shown to be alleviated by the addition of carboxymethyl cellulose (CMC). Thus, leguminous proteins’ distinct qualities imply that they have a great deal of potential as a resource for widespread use in the food business^78^. According to^87^, adding legume flour (lentil, chickpea, and yellow pea) to gluten-free rice pasta enhanced cooking quality by reducing cooking loss (less than 6.0%). These findings are consistent with their findings.

### Color attributes of cooked pasta

**Fig. 5:**
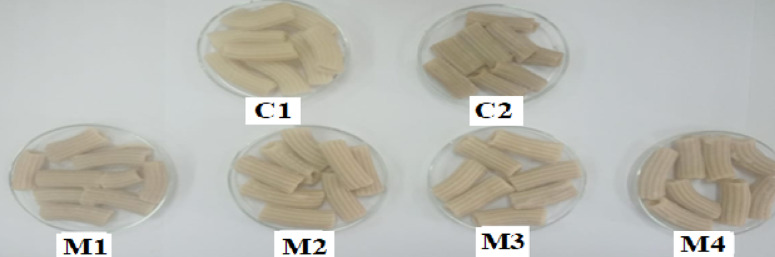
Photograph of cooked pasta.

Table 11 shows the color characteristics of various cooked pasta samples (pasta photographs are shown in Fig. 5), quantified through lightness (L*), redness (a*), and yellowness (b*). Compared with the other samples, the rice pasta (C1) presented the highest lightness value (L* = 90.09), with significant differences (*P* ≤ 0.05), indicating a notably lighter hue relative to the other samples. Conversely, the oat pasta (C2) presented significantly lower lightness (L* = 70.48), indicative of darker coloration, which aligns with the typical visual traits of oat-based pasta. As reported by^76^, more reddish-colored noodles were observed in comparison with the control noodles (100% wheat flour), a phenomenon that aligns with the findings of^88^. A significant distinction in the visual characteristics of the prepared noodles was noted at incorporation levels of 20% and above, where the color appeared darker and the texture exhibited increased roughness relative to the control.

**Table 11:** Color attributes of cooked pasta.

Pasta samples	Color parameters		
	L* (lightness)	a* (redness)	b* (yellowness)
C1	90.09^a^ ± 1.37	0.10^e^ ± 0.01	4.28^e^ ± 0.09
C2	70.48^e^ ± 0.56	1.74^c^ ± 0.09	11.74^c^ ± 0.09
M1	73.96^d^ ± 1.19	1.08^d^ ± 0.03	9.31^d^ ± 0.28
M2	77.29^c^ ± 0.26	3.74^b^ ± 0.09	15.40^b^ ± 0.48
M3	79.34^c^ ± 0.47	5.56^a^ ± 0.30	17.27^b^ ± 0.38
M4	82.83^b^ ± 0.25	5.97^a^ ± 0.10	19.89^a^ ± 1.10
LSD at 0.05	3.233	0.563	2.115

Means (*n* = 3) in the same column with different small letters are significantly different (*P* < 0.05).

This roughness may be attributed to the elevated fiber content and the irregularities encountered during the noodle preparation process. The redness values (a*) were minimal in C1 (0.10), suggesting a lack of red tint, whereas samples with higher LF contents (M3 and M4) presented the highest redness values (5.56 and 5.97, respectively), indicating a significant increase in redness and a more pronounced red shade as LF was added^87^. reported the same findings. Similarly, the lentil-enhanced pasta formulations had a more noticeable yellowish tone, as seen by the yellowness values (b*), which peaked in M4 (19.89) and were lowest in C1 (4.28). These differences in color measurements suggest that adding LF darkens the pasta while also intensifying its red and yellow color, which is probably due to the natural pigments in LF.

All together, the results highlight how LF significantly affects the color characteristics of cooked pasta, making it more vibrant and more translucent than rice-oat pasta. In general, a rise in phenolic and protein compounds is associated with lower L* values^69^. LF’s higher protein and phenolic content may affect its color properties, causing cookies prepared with LF to brown more noticeably than those made with wheat flour. Additionally^89^, found a strong correlation between color characteristics and phenolic chemicals^14^. observed that cookies baked with C. album flour, which has a high protein and phenolic component content, had a darker coloration. However, in this case the dark color of the oat-based pasta may be due to the oat flour and the lack of a technology that can effectively separate the flour from the bran, as flour color depends largely on the color of the oat hull. Therefore, lentil flour helped reduce the dark color, despite its high protein and phenolic content.

### Organoleptic characteristics of cooked pasta

**Fig. 6:**
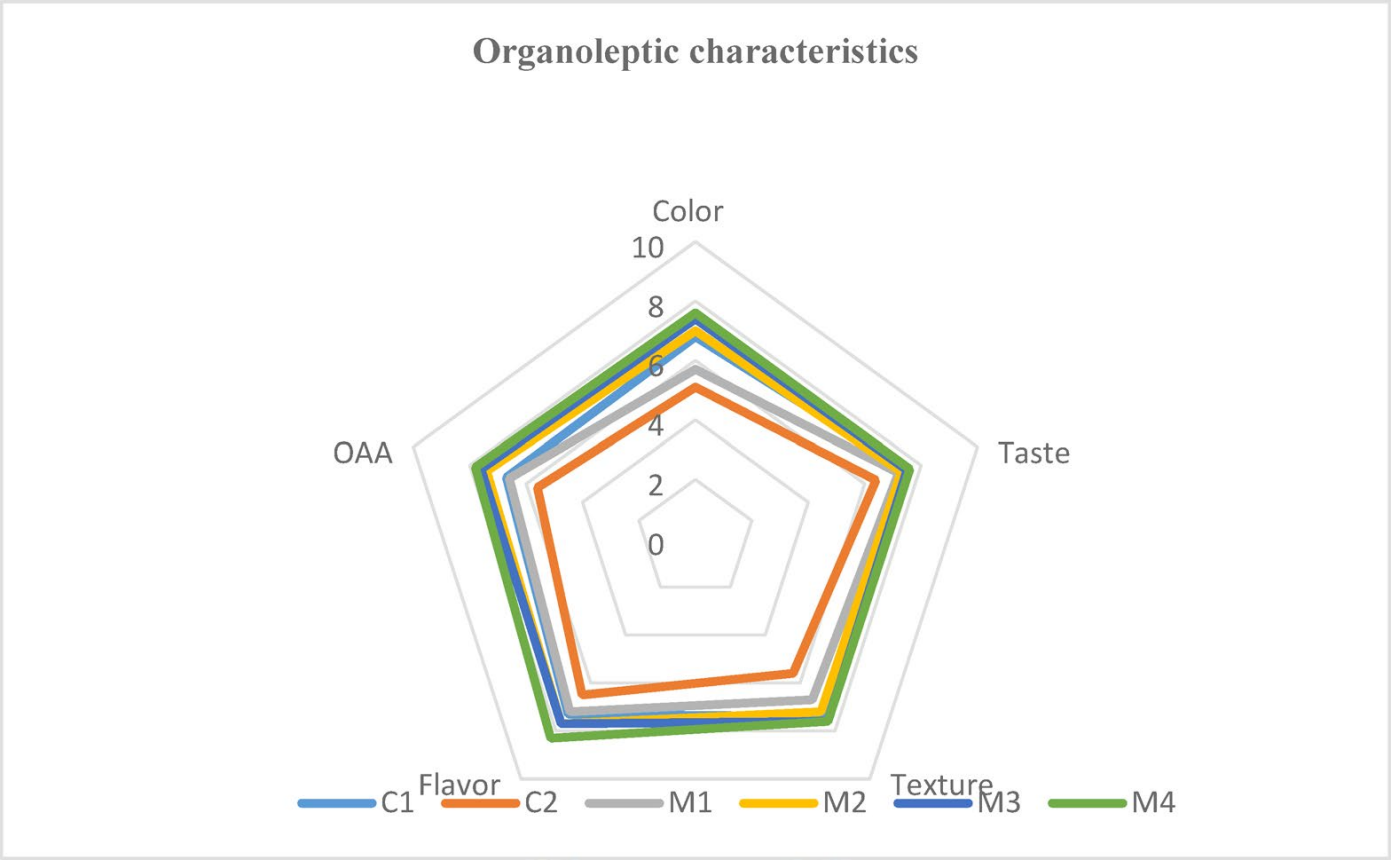
Organoleptic characteristics of cooked pasta.

The organoleptic characteristics of various pasta formulations are evaluated using a verbal descriptor scale from “like extremely” to “dislike extremely” on a scale of 1 to 9 (Fig. 6). Completely made of RF, rice pasta (C1) received the highest ratings for flavor (7.30), texture (7.40), and taste (7.50), all of which fell into the “like moderately” range. However, it received a lower score for color (6.80), reflecting a “like slightly” preference.

Overall acceptability (OAA) for C1 was 6.70, which indicates a moderate level of liking (“like slightly”). On the other hand, oat pasta (C2), which is made entirely of OAF, scored the lowest for all sensory attributes, especially color (5.10, “neither like nor dislike”) and flavor (6.50, “like slightly”), with an OAA of 5.60, which indicates a slightly negative acceptance (“dislike slightly”). The incorporation of LF into the rice-oat pasta blends (M2 to M4) led to notable improvements in their sensory characteristics. Thus, the M4 sample (35% RF, 35% OAF, and 30% LF) achieved the highest ratings in all categories, with flavor reaching 8.30 (“like very much”) and an OAA of 7.80 (“like very much”). These findings indicate that the addition of LF significantly enhances the sensory quality of pasta, with M4 emerging as the most favored formulation in terms of color, taste, texture, flavor, and overall acceptability.

The inclusion of LF not only increases the nutritional profile of the pasta but also increases its appeal to consumers. These results are in line with those of^87^, who looked into the effects of adding legumes to gluten-free rice pasta and found that adding LF improves taste, color, and overall acceptability, while others pointed out that adding lentils to cereal-based products may lead to a rise in the consumption of these foods. LF has traditionally been employed in cake production to increase both nutritional value and sensory characteristics^90^.

## Conclusion

RSM was employed to optimize the concentrations of xanthan gum (XG) and guar gum (GG) in the formulation of gluten-free pasta-based rice flour to increase the hardness of dry pasta. The application of the ideal proportion of binding materials in the production of gluten-free pasta resulted in the highest degree of hardness compared to pasta made from oat flour. The optimal formulation parameters identified through RSM were 2.00% XG and 0.75% GG. As a result, the predicted responses for the hardness of dry gluten-free pasta were deemed satisfactory. The addition of lentil flour had a negative effect on hardness, which gradually decreased with increasing proportion of lentil flour. On the other hand, the addition of lentil flour improved the functional properties and protein digestibility, in addition to improving the nutritional quality in general. These findings provide a feasible formulation technique for creating nutritionally enriched gluten-free pasta with acceptable texture and functional characteristics, benefiting individuals with celiac disease or those pursuing gluten-free options. Subsequent inquiries should focus on amplifying the optimal formulation for industrial manufacturing, encompassing drying parameters. Exploring other legume flours or dietary fibers as functional ingredients could also help improve both textural and nutritional characteristics without compromising product quality.

## Data Availability

The datasets used and/or analysed during the current study available from the corresponding author on reasonable request.
